# Identification of key genes affecting intramuscular fat deposition in pigs using machine learning models

**DOI:** 10.3389/fgene.2024.1503148

**Published:** 2025-01-06

**Authors:** Yumei Shi, Xini Wang, Shaokang Chen, Yanhui Zhao, Yan Wang, Xihui Sheng, Xiaolong Qi, Lei Zhou, Yu Feng, Jianfeng Liu, Chuduan Wang, Kai Xing

**Affiliations:** ^1^ College of Animal Science and Technology, China Agricultural University, Beijing, China; ^2^ College of Animal Science and Technology, Beijing University of Agriculture, Beijing, China; ^3^ Beijing Animal Husbandry Station, Beijing, China

**Keywords:** machine learning, pig, transcriptome, intramuscular fat, key genes

## Abstract

Intramuscular fat (IMF) is an important indicator for evaluating meat quality. Transcriptome sequencing (RNA-seq) is widely used for the study of IMF deposition. Machine learning (ML) is a new big data fitting method that can effectively fit complex data, accurately identify samples and genes, and it plays an important role in omics research. Therefore, this study aimed to analyze RNA-seq data by ML method to identify differentially expressed genes (DEGs) affecting IMF deposition in pigs. In this study, a total of 74 RNA-seq data from muscle tissue samples were used. A total of 155 DEGs were identified using a limma package between the two groups. 100 and 11 significant genes were identified by support vector machine recursive feature elimination (SVM-RFE) and random forest (RF) models, respectively. A total of six intersecting genes were in both models. KEGG pathway enrichment analysis of the intersecting genes revealed that these genes were enriched in pathways associated with lipid deposition. These pathways include α-linolenic acid metabolism, linoleic acid metabolism, ether lipid metabolism, arachidonic acid metabolism, and glycerophospholipid metabolism. Four key genes affecting intramuscular fat deposition, *PLA2G6, MPV17, NUDT2*, and *ND4L*, were identified based on significant pathways. The results of this study are important for the elucidation of the molecular regulatory mechanism of intramuscular fat deposition and the effective improvement of IMF content in pigs.

## Introduction

Intramuscular fat is one of the most important determinants of pork quality (Zhang et al., 2021) and affects the sensory qualities of pork, such as tenderness, flavor, and juiciness (Fernandez et al., 1999). Intramuscular fat content is influenced by several factors (Malgwi et al., 2022), among which genetic factors play a decisive role in intramuscular fat content (Hamill et al., 2012). The genes that have been studied and found to affect intramuscular fat deposition are *ROBO2* (Sato et al., 2017), *HS6ST3* (Jiang et al., 2011), *PLIN5* (Puig-Oliveras et al., 2014) and *NR4A1* (Qin et al., 2018), and so on.

RNA-seq technology is widely used in the field of genetic breeding in livestock production. In the field of animal husbandry, numerous studies have utilized transcriptomics to uncover the intrinsic connection between gene expression and economic traits. For instance, researchers have revealed the rules of muscle development during the embryonic stage of Chengkou pheasants through transcriptomic analysis (Ren et al., 2021); identified the potential regulatory genes associated with heat tolerance in Holstein dairy cows (Liu et al., 2020); and determined the genes related to the growth and development of skeletal muscles by comparing the transcriptomic differences among different duck breast muscle tissues and among different pigeon breast muscle tissues (Wang Z. et al., 2021; Ding et al., 2021). In recent years, there have been many reports on transcriptomic studies of traits related to intramuscular fat deposition in pigs by RNA-seq technology. Li et al. analyzed transcriptomic data from the longissimus dorsi muscle (LDM) of Wei and Yorkshire pigs and found that many differentially expressed lncRNAs may influence the developmental process of IMF by regulating its potential target genes (Li et al., 2020). Cho et al. compared IMF in western and Korean native pig breeds with LDM and identified the *MYH3* on pig chromosome 12 as a causal gene affecting intramuscular fat deposition, which can inhibit myogenic regulatory factor binding and thus promote intramuscular fat deposition through a structural variation of 6-bp deletion on the promoter (Cho et al., 2019). Huang et al. analyzed IMF using Laiwu pig and Large White pig and identified a total of 513 mRNAs and 55 lncRNAs differentially expressed between the two pig breeds and identified 31 key lncRNAs by co-expression network construction and cis- and trans-regulated target gene analysis (Huang W. et al., 2018). Through transcriptomic studies, several candidate genes have been identified to affect the process of intramuscular fat deposition in pigs, such as *LEP* (Li et al., 2010), *FASN* (Crespo-Piazuelo et al., 2020) *ACACA* (Piórkowska et al., 2020), and so on. Although the transcriptome provides an efficient tool for the genetic resolution of important traits, transcriptome sequencing analysis is difficult for later functional validation and has a high false positive rate due to the small sample size. Current transcriptome data analysis methods mainly focus on the processing of a small number of samples from a single experiment, and the data from different samples cannot be integrated, which is not deep enough for data mining. Gene expression exhibits temporal specificity and spatial specificity. Spatial specificity implies that in multicellular organisms at specific growth and development stages, the same gene is expressed differently in various tissues and organs. The spatial distributional differences manifested by gene expression along the sequence of time or stage are actually determined by the distribution of cells in organs. Hence, the spatial specificity of gene expression is also known as cell specificity or tissue specificity. Due to the significant influence of both space and time on gene expression and the considerable variations in the samples employed in different studies, it becomes challenging to discover the major effector genes that universally regulate fat deposition.

ML, as an important component in the field of artificial intelligence, provides a new strategy for the study of histology. Currently, the method has been widely used in many areas of multi-omics research (Hashimoto et al., 2020; Lee et al., 2021). The classification function of ML in cancer genome classification or typing can be used to discover new biomarkers, new drug targets, and a deep understanding of cancer-induced genes (Huang S. et al., 2018). They have also been applied to genome selection in animal husbandry and have slightly improved their accuracy compared to traditional methods (Waldmann et al., 2020). For transcriptomic data, the large number of expressed genes determines the high complexity of the model, and ML, a new big data fitting method, can effectively fit complex data and accurately identify samples and genes (Waldmann et al., 2020). In addition, the small number of individual study samples affects the accuracy of machine learning analysis; therefore, multiple datasets need to be integrated to accurately predict and mine key genes with machine learning algorithms. SVM-RFE effectively reduces the feature dimension through recursive feature elimination and is suitable for high-dimensional small sample data. RF offers gene importance scores, can capture nonlinear relationships and feature interactions, and demonstrates robustness against noise and outliers. By contrast, KNN, K-means, neural networks, and naive Bayes are not appropriate for feature selection: KNN lacks a feature evaluation mechanism; K-means is not suitable for identifying differential genes; neural networks require a large quantity of data; and naive Bayes assumes feature independence, which is inconsistent with the characteristics of gene data (Sheth et al., 2022). In this study, the two methods of SVM-RFE and RF were chosen to screen differentially expressed genes mainly because they possess certain advantages in feature selection and handling high-dimensional data.

Therefore, this study collected the longissimus dorsi muscle tissue samples transcriptome datasets from pigs with different IMF content including our study and NCBI’s Sequence Read Archive (SRA) database. Two machine learning methods RF and SVM-RFE were used for identifying key genes affecting IMF content. The findings are helpful for further exploring the molecular regulatory mechanisms of intramuscular fat deposition in pigs.

## Materials and methods

### Acquisition of transcriptome sequencing data

In this study, 53 Songliao Black sows and 132 Long White sows were selected from the Tianjin Ninghe Original Breeding Pig Farm. These pigs were reared under the identical feeding conditions. When the pigs were raised to approximately 100 kg, the backfat thickness was determined using the HONGDA HS-1500 veterinary B ultrasound machine (between the second-to-last and fourth ribs, 5 cm from the dorsal midline) (Suzuki et al., 2009). To avoid the influence of different genetic backgrounds, three pairs of individuals from each breed with extreme differences in backfat thickness were slaughtered and the longissimus dorsi muscle tissues were collected. One portion was analyzed for the IMF content of the samples using the FOSSDSCAN near-infrared rapid analyzer for food components, while the other portion was preserved in liquid nitrogen for RNA extraction.

Total RNA was extracted from the longissimus dorsi muscle tissue using the Trizol kit according to the product instructions, and a total of 12 samples were extracted. The extracted RNA was diluted with 1% DEPC water and denatured for 2 min at 70°C. The quality of the RNA was checked by Agilent 2100, and the library was constructed by Illumina TruSeqTM RNA kit. The constructed libraries were sequenced by the Illumina Hiseq 2000 sequencing platform with pair ends (PE). In this study, eight datasets were also downloaded from the SRA database (https://www.ncbi.nlm.nih.gov/sra/) under NCBI, namely PRJNA776032, PRJNA302287, PRJNA359473, PRJNA480676, PRJNA695218, PRJNA387276, PRJNA743884, and PRJNA604841. A total of 62 samples with an equal number of samples in high and low intramuscular fat groups in each dataset, including muscle tissue samples from Min, Wannanhua, Diannan Small-ear, Tibetan, Landrace, Large White, Iberian, Nanyang Black, Wei, and Dingyuan pigs.

A total of 74 samples were collected and these data were processed by the same method, and the raw data were quality-controlled using fastp software (Chen et al., 2018) to remove sequences with connectors and low-quality sequences (reads with Q ≤ 20). High-quality sequences were aligned to the pig reference genome *Sus scrofa* 11.1 using HISAT2 software (Kim et al., 2019) and annotated, and the expression of genes in different samples was calculated by HTSeq software (Anders et al., 2015). After obtaining gene expression profiles all data sets were integrated and samples were grouped according to phenotypic indicators (backfat thickness and intramuscular fat content) (Table 1). The downloaded data categorized lean pigs as the high IMF group and local pigs as the low IMF group.

**TABLE 1:** Sample information from different datasets.

Accession number	Breed	Day	Tissue	HIMF group	LIMF group	Sex	Reference
Ours(PRJNA1043865)	Landrace, Song liao black pig	—	muscle	6	6	F	—
PRJNA776032	Large White × Min pig	240	muscle	5	5	M, F	Cheng et al. (2021)
PRJNA302287	Yorkshire, Wannanhua	150	muscle	3	3	F	Li et al. (2016)
PRJNA359473	Diannan Small-ear pig, Tibetan pig, Landrace, Yorkshire	180	muscle	2	2	—	Wang et al. (2015)
PRJNA480676	Iberian purebred pig	500	muscle	6	6	M	Muñoz et al. (2018)
PRJNA695218	Nanyang black pig	180	muscle	3	3	F	Wang L. et al. (2021)
PRJNA387276	Yorkshire, Wei pig	150	muscle	3	3	F	Xu et al. (2018)
PRJNA743884	Ding yuan pig	300	muscle	3	3	F	Zhang et al. (2022)
PRJNA604841	Italian Large White pig	240	muscle	6	6	M, F	Zappaterra et al. (2020)

Note: HIMF, stands for the high intramuscular fat group, and LIMF, stands for the low intramuscular fat group; F denotes sows, and M denotes gilts.

### Data pre-processing

To make the data comparable across studies, all data were converted to fragments per thousand bases of transcripts per million mapped reads (FPKM). The genes were screened with the following criteria: (1) removal of genes without symbol names; (2) removal of genes expressed in less than 10 samples. Before analyzing the data this study adjusted for batch effect, processed by the combat function of the sva package of the R-4.2.2 package, and visualized the gene expression data before and after the batch effect adjustment. Sva is a commonly used batch effect adjustment method that removes the batch effect by identifying and adjusting for potential influencing factors while preserving the biological differences in the data and avoiding biological conclusions.

### Differential expression gene extraction

In this study, differential expression analysis was performed using the algorithm provided by the limma program package of the R-4.2.2 software packages (Ritchie et al., 2015). The data of the high intramuscular fat group was compared with the low intramuscular fat group, and the data were screened at P < 0.05, |log_2_ FC| > 1 to select genes with significance. The occurrence of false positives in differential expression analysis was controlled in our study by adjusting the batch effect with the ComBat function. The DEGs were visualized by volcano plot. The samples were clustered using DEGs through the Microsign online analysis cloud platform (www.bioinformatics.com.cn).

### Construction of machine learning models

**FIGURE 1:**
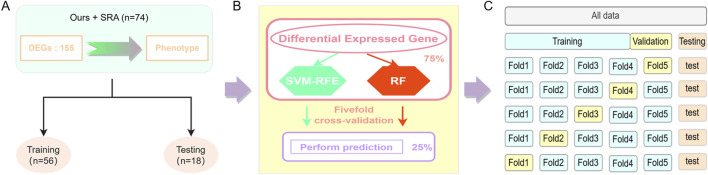
Model construction. **(A)** Data set division, **(B)** Classifier construction, **(C)** Fivefold cross-validation.

To further identify the candidate genes affecting intramuscular fat deposition in pigs, machine-learning models were constructed based on the results of differential expression analysis. The expression levels of each DEG were scaled to the [0–1] interval using the maximum-minimum normalization method, to unify the weights of features and improve model accuracy. The data set is divided into a training set and a validation set with 74 samples, of which 75% of the samples were used as the training set to build the model, and the remaining 25% were used as the validation set to validate the performance of the model (Figures 1A, B). Two supervised learning classifiers, including SVM-RFE (Sahran et al., 2018)and RF (Zhao et al., 2018) models, were tested in this study. The e1071 program package of the R-4.2.2 package (https://cran.r-project.org/web/packages/e1071/index.html) was used to implement SVM-RFE for differentially expressed gene screening, while RF was done using the randomForest program package (https://www.stat.berkeley.edu/∼breiman/RandomForests/). To avoid overfitting the constructed models, the models were validated using a fivefold cross-validation to adjust the suitable parameters (Figure 1C).

### Biological function analysis

To understand the functions of the genes screened by the machine learning model, biological functional analysis and their visualization were performed. Kyoto Encyclopedia of Genes and Genomes (KEGG) pathway enrichment analysis of the identified significant genes was performed through Omicshare Kidio Bioinformatics Cloud Platform (https://www.omicshare.com/).

## Results

### Sequencing quality assessment

By analyzing the quality of the raw sequencing data, it was found that the data quality was all as expected (Additional file 1: Supplementary Table S1). The quality-controlled high-quality reads were compared to the reference genome of pigs, and the mapping rates were found to be above 90% (Additional file 2: Supplementary Table S2). The data are reliable and can be analyzed in the next step.

### Batch effect adjustment

**FIGURE 2:**
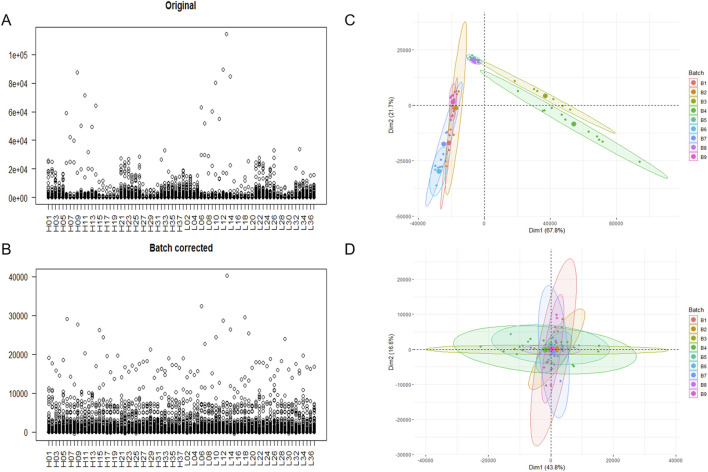
Mapping before and after removal of the batch effect. **(A)** Box line plot before batch effect adjustment. **(B)** Box line plot after batch effect adjustment. **(C)** PCA plot before batch effect adjustment. **(D)** PCA plot after batch effect adjustment.

The initially obtained gene expression profiles had a total of 31,908 genes, and after retaining the genes with symbol names and those expressed in at least 10 samples, 9,675 genes remained. The remaining data were subjected to the batch effect adjustment, and the box plot shows that the range of gene expression values in the samples decreased after the batch effect adjustment, indicating a reduction in outliers (Figures 2A, B). After principal component analysis, it was found that before the batch effect adjustment, the samples were divided into three groups, indicating heterogeneity among the samples, and after the batch effect adjustment. The samples clustered together, indicating similarity among the samples (Figures 2C, D).

**FIGURE 3:**
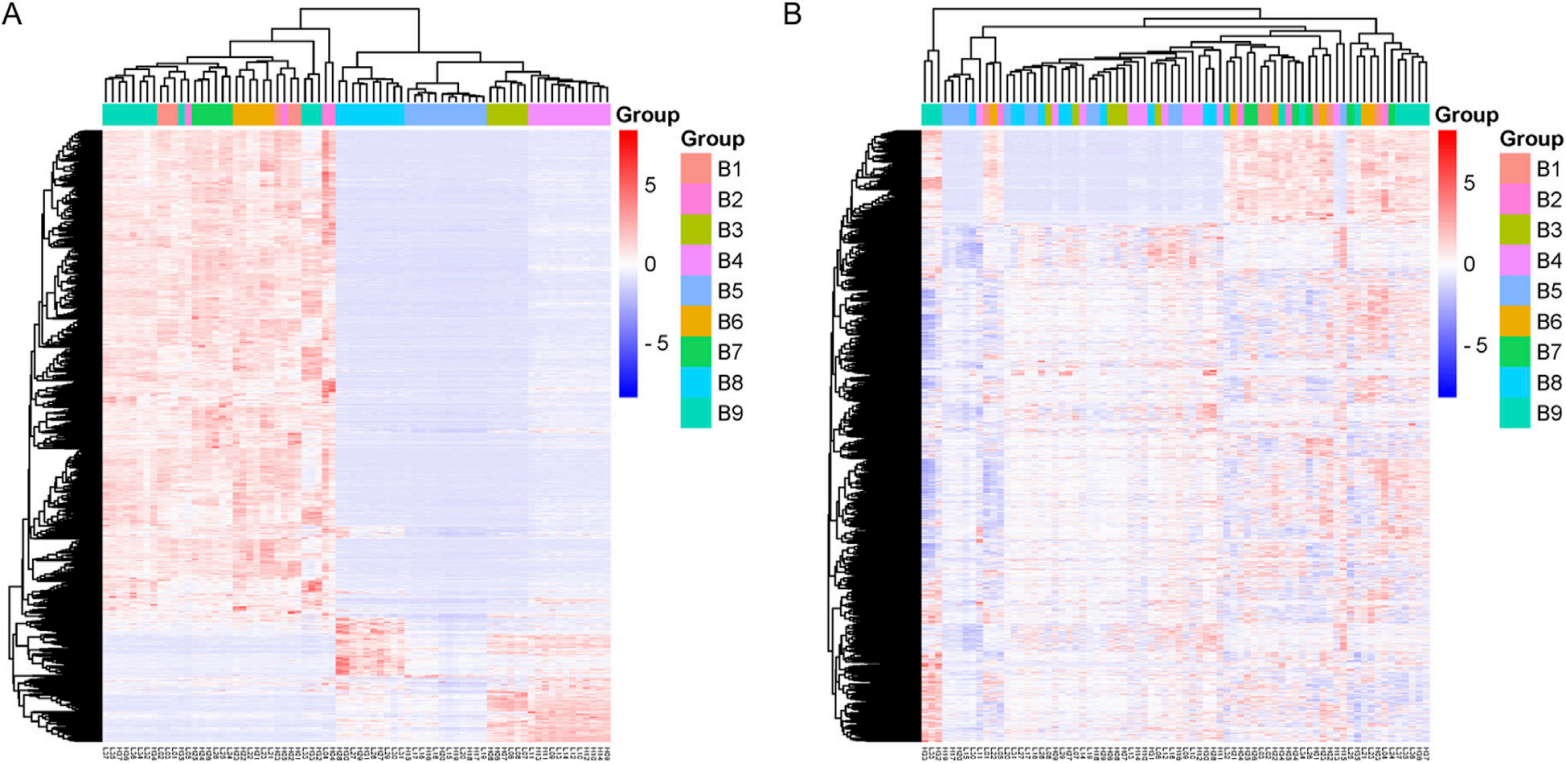
Sample clustering heat map. **(A)** The heat map of clustering before batch effect adjustment. **(B)** The heat map of clustering after batch effect adjustment.

The sample clustering heat map further showed that the samples were more homogeneous after adjusting the batch effect (Figure 3).

### Analysis of DEGs

The limma package was used to perform differential expression analysis on the nine datasets, and 180, 1,526, 315, 365, 1,097, 570, 1,452, 452, and 358 genes were identified, respectively. No common differential genes were found among these datasets (Supplementary F S1). This indicates that it is difficult to find genes that regulate fat deposition with generalizability by aggregating DEGs between different datasets.

**FIGURE 4:**
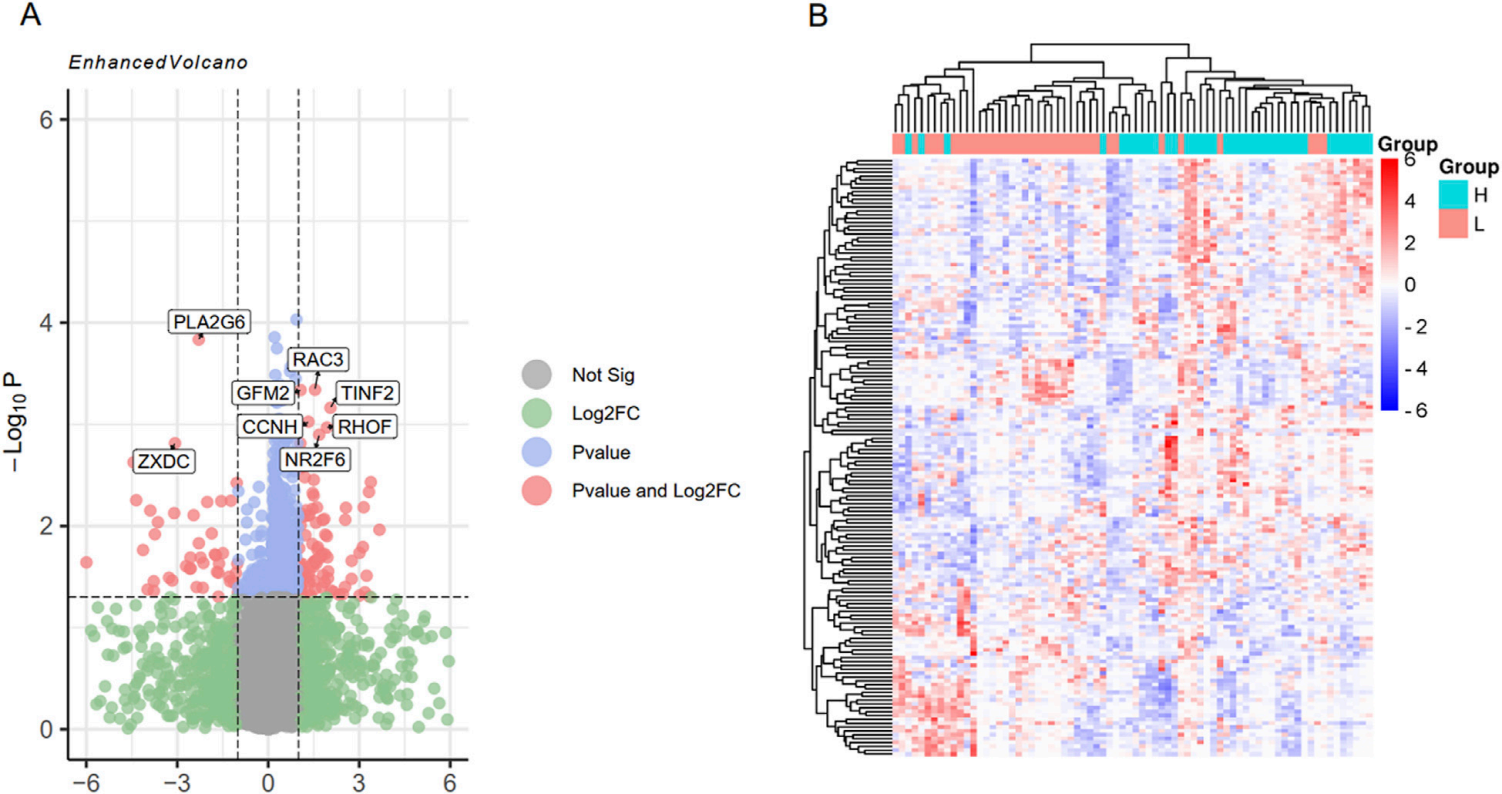
Differential expression analysis of the integrated data set. **(A)** represents a volcano plot of DEGs, which shows the eight genes with the most significant P values; **(B)** represents the sample clustering heat map of DEGs.

Using the limma package, differential expression analysis was performed on the integrated dataset, and 155 DEGs were screened. Among them, 99 genes were highly expressed in the high intramuscular fat group, and 56 genes were highly expressed in the low intramuscular fat group (Figure 4A). In addition, these screened genes can effectively separate the high intramuscular fat group from the low intramuscular fat group (Figure 4B).

### Feature selection

**FIGURE 5:**
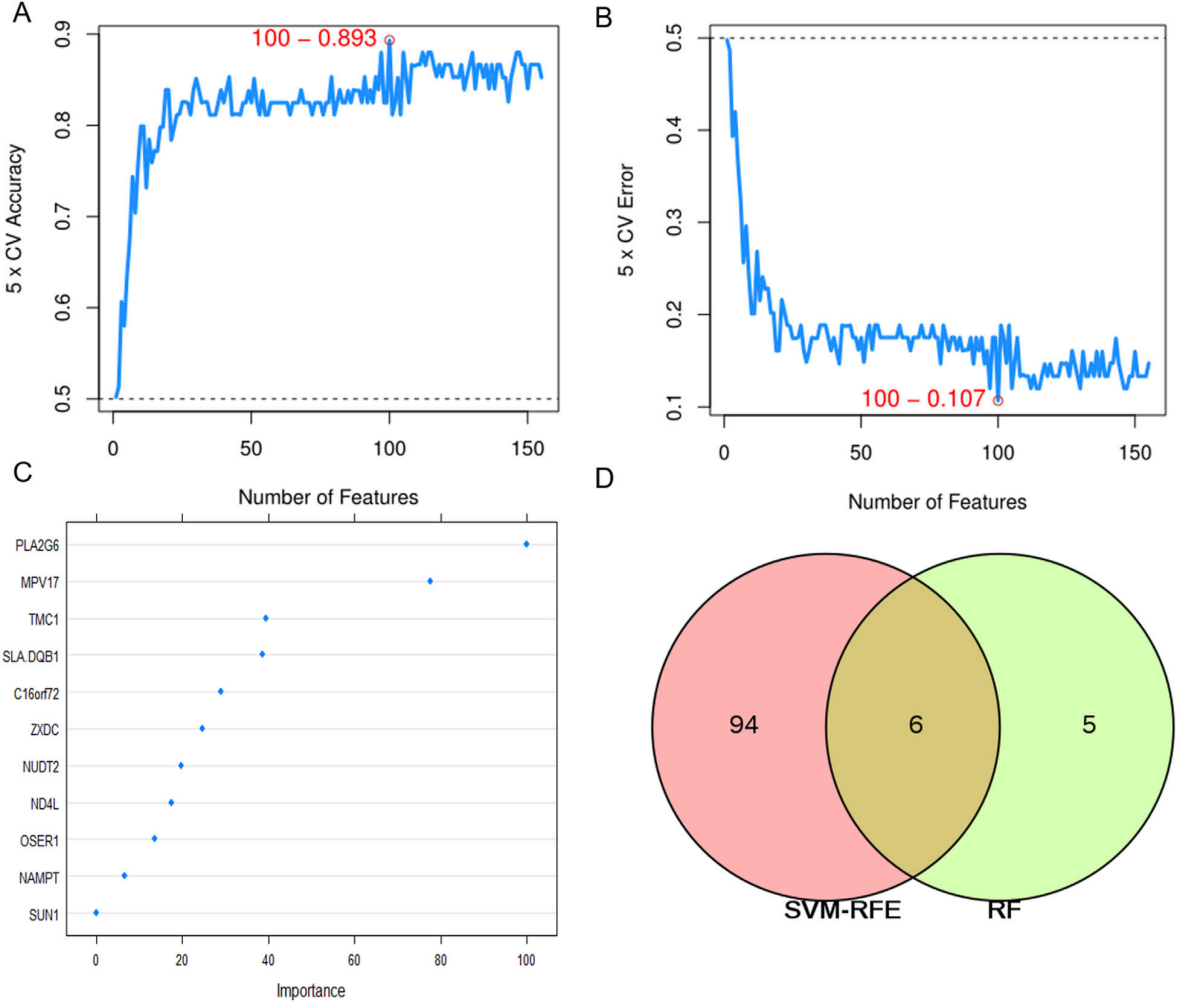
Two algorithms are used for feature selection. **(A)** the accuracy of the SVM-RFE model. **(B)** error rate of SVM-RFE model. **(C)** importance ranking of genes identified by random forest. **(D)** The intersection feature selection between SVM-RFE and RF algorithms.

The SVM-RFE model screened 100 significant genes (Additional file 3: Supplementary Table S3), RF screened 11 significant genes, and Table 2 shows the top 15 ranked genes screened by the SVM-RFE model. A total of six common important features were screened by both models (Figure 5). Area Under Curve (AUC) is defined as the area beneath the Receiver Operating Characteristic (ROC) Curve. Given that the ROC curve is typically located above the line y = x, the range of AUC values lies between 0.5 and 1. The AUC value is equivalent to the probability that a randomly chosen positive example is ranked higher than a randomly chosen negative example (Fawcett, 2006). Thus, the larger the AUC value, the more likely the current classification algorithm is to rank the positive sample before the negative sample, indicating a better classification performance.

$$AUC=\sum_{i-2}^{m}\frac{\left(x_{i}-x_{i-1}\right)*(y_{i}+y_{i-1})}{2}$$

**TABLE 2:** The top15 feature vectors of the support vector machine model.

FeatureName	FeatureID	AvgRank
*SUN1*	37	8.6
*ETFRF1*	128	11.4
*RPS4X*	62	12.2
*ZXDC*	6	16
*ANXA11*	50	16
*CTSZ*	36	20
*SMAD3*	22	22.6
*KCNAB1*	102	26
*ID1*	142	26.8
*MRPL15*	116	27.2
*CCNH*	72	27.4
*XIRP1*	132	28.2
*LYL1*	101	29
*EIF3M*	65	29.4
*GGCX*	108	32.2

Note: This table shows the top 15 genes, with the first column indicating the feature name, the second column indicating the feature ID, and the third column indicating the average ranking coefficient; the smaller the coefficient, the more important the feature is.

Visualized by ROC curves, AUC of SVM-RFE and RF are 0.893 and 0.86, respectively (Supplementary Figure S2), indicating that the former technique is superior to the latter.

In addition, this study identified 10 genes associated with fat deposition from the 100 genes screened by SVM-RFE, namely *APP, CTSZ, EIF4EBP1, FABP4, FAM184B, ID1, PLA2G6, SELENOF, SRGN,* and *TSPO*, and these genes are associated with fat deposition (Table 3).

**TABLE 3:** Fat deposition-related DEGs.

Gene symbol	Gene description	Gene function	Reference
*APP*	The amyloid beta precursor protein	Correlated with the level of cytokine expression in adipocytes	Lee et al. (2008)
*CTSZ*	Cathepsin Z	Fat deposition process in pigs	Russo et al. (2008)
*EIF4EBP1*	Eukaryotic translation initiation factor 4E binding protein 1	Involved in adipose tissue development	Tsukiyama-Kohara et al. (2001)
*FABP4*	Fatty acid binding protein 4	Transport of long-chain fatty acids	Zhou et al. (2010)
*FAM184B*	Family with sequence similarity 184 member B	Correlation with fatty acid content	Yuan et al. (2021)
*ID1*	Inhibitor of DNA binding 1	Expressed in brown fat and white fat	Patil et al. (2017)
*PLA2G6*	Phospholipase A2 group VI	Catalyzing the hydrolysis of fatty acids in glycerophospholipids	Alecu and Bennett (2019)
*SELENOF*	Selenoprotein F	Involved in lipid metabolic processes	Zheng et al. (2020a)
*SRGN*	Serglycin	Highly expressed in adipocytes	Savedoroudi et al. (2019)
*TSPO*	Translocator protein	Regulation of lipid metabolism	Kim et al. (2020)

**FIGURE 6:**
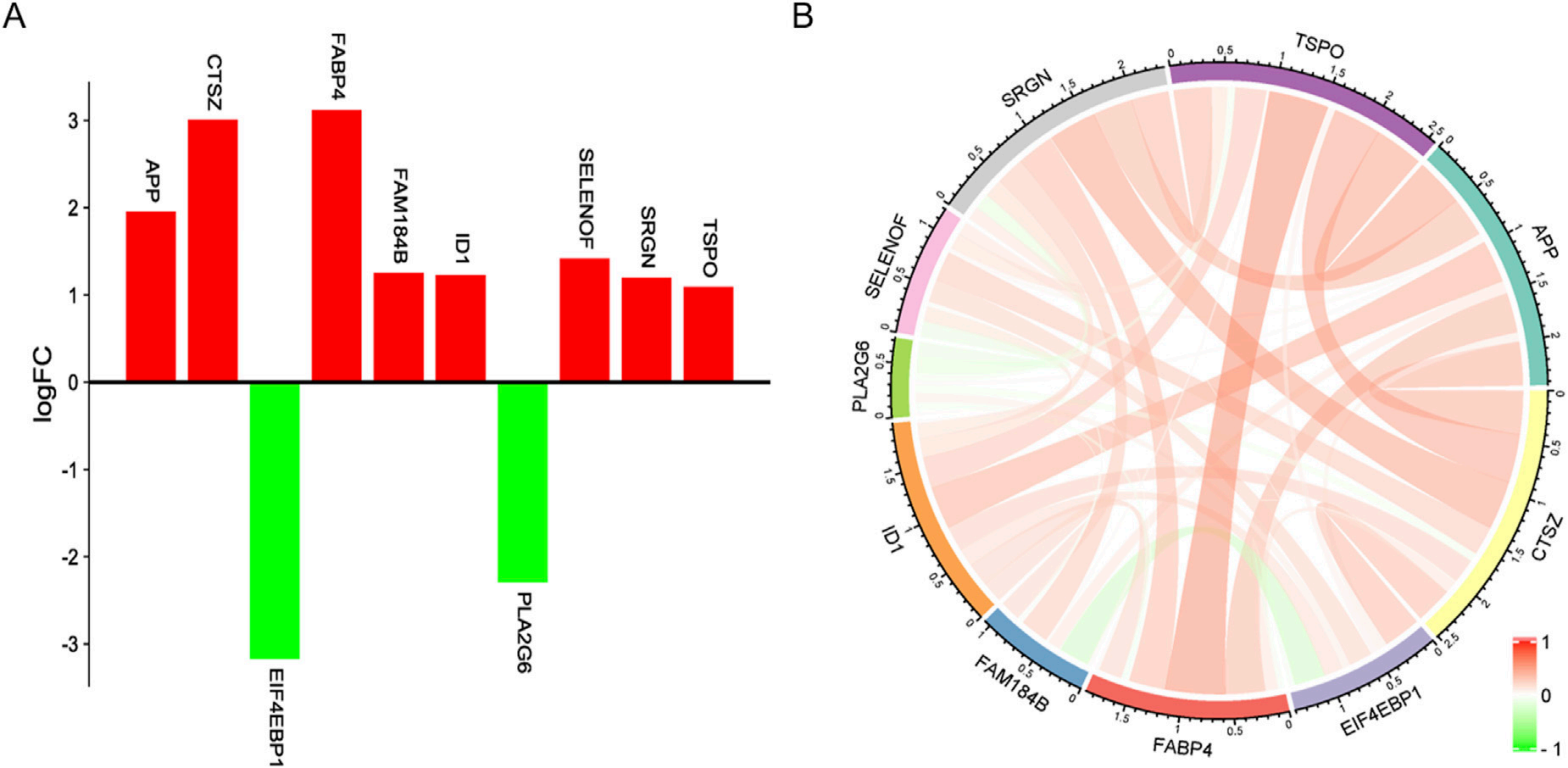
Gene expression profile. **(A)** Red represents upregulation and green represents downregulation. **(B)** The color, and width of the ribbon correlate with the correlation of gene expression, where red indicates a positive correlation and green indicates a negative correlation.

Among them, eight genes were highly expressed in the high intramuscular fat group compared to the low intramuscular fat group, and only *EIF4EBP1HE* and *PLA2G6* were highly expressed in the low intramuscular fat group. Moreover, there was mainly a positive correlation between these genes (Figure 6).

### Sample distribution

**FIGURE 7:**
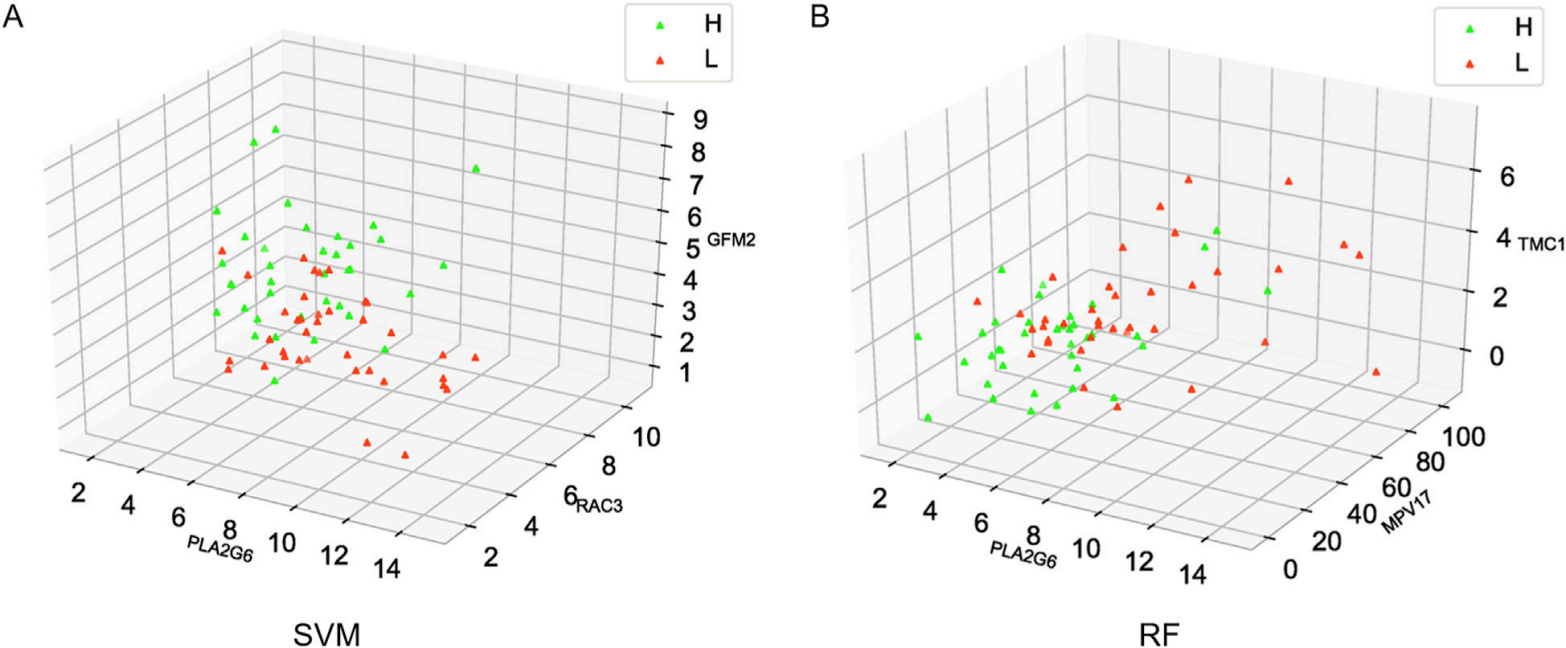
Distribution of high and low group samples. **(A)** Distribution of SVM-RFE samples, **(B)** Distribution of RF samples.

To visualize the distribution of samples in the high intramuscular fat group and the low intramuscular fat group, the distribution of samples was visualized using a 3D scatter plot. The green triangles in Figure 7 represent the high intramuscular fat group and the red triangles represent the low intramuscular fat group, and the top three most important genes were selected as coordinates. It can be seen from the figure that the distribution of the two groups of samples is very different (Additional file 4: Supplementary Table S4), and therefore, the model this study constructed can effectively distinguish the high intramuscular fat group from the low intramuscular fat group. (Figure 7).

### Pathway enrichment analysis of intersection genes

**FIGURE 8:**
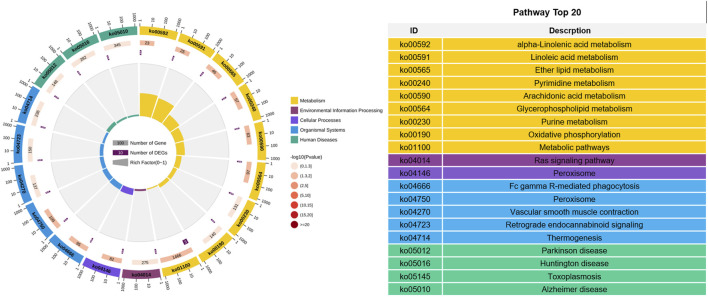
KEGG pathway analysis.

Six intersecting genes screened using two models were subjected to KEGG pathway enrichment analysis, and it was found that these genes were enriched in a total of 20 pathways. Among them, there are 10 significantly enriched pathways, and most of them are related to fat deposition, such as α- Linoleic acid metabolism, linoleic acid metabolism, ether lipid metabolism, glycerophospholipid metabolism, and arachidonic acid metabolism, etc. (Figure 8). Four genes related to fat deposition were screened based on significant pathways, namely *PLA2G6*, *MPV17*, *NUDT2*, and *ND4L*.

**FIGURE 9:**
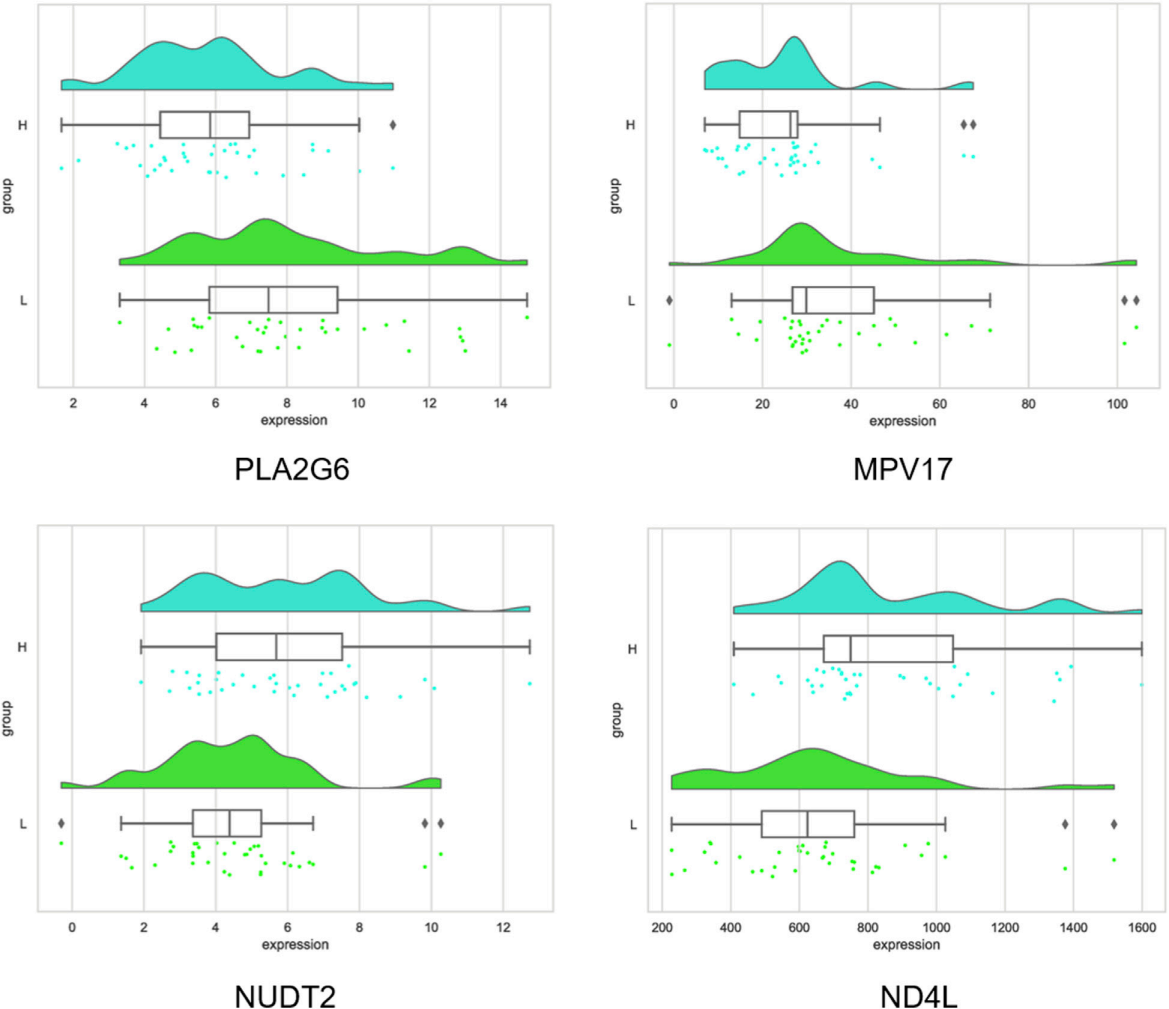
Distribution of important gene expression values.

The four important genes were *PLA2G6, MPV17, NUDT2*, and *ND4L*, where *PLA2G6* and *MPV17* were upregulated in the high intramuscular fat group, and *NUDT2* and *ND4L* were downregulated in the high intramuscular fat group compared to the low intramuscular fat group (Figure 9).

## Discussion

The integration of data from different transcriptomic studies is important for improving the reliability and generalizability of the results, allowing access to valid information that is not available from individual studies (Lazar et al., 2013; Mooney and Mcweeney, 2014). In our study, this was confirmed by screening the DEGs in each of the nine datasets using traditional differential analysis methods, and as a result, no common gene was found in these datasets. In contrast, when this study integrated multiple transcriptomic datasets for differential expression analysis, a common set of DEGs was found, and the results of this study are biologically significant.

When integrating the dataset, the batch effect needs to be adjusted to unify the data from different studies. This is because the data this study acquired may lead to errors due to differences in sample collection time, sequencing platform and pig breed, tissue, age and sex, and so on. So that the DEGs this study eventually found are not the genes that differ, resulting in false positives.

In this study, the large dataset was initially screened by traditional variance analysis methods, and then machine learning algorithms were utilized to further identify DEGs. A total of two classification algorithms, SVM-RFE and RF, were trained, and a set of key predictors was obtained for each classifier. The intersection of important genes was screened by these classifiers and functional annotation of these genes yielded key candidate genes affecting fat deposition. This study finally screened a total of four important genes, *PLA2G6, MPV17, NUDT2,* and *ND4L. PLA2G6* is a lipid regulator that catalyzes the hydrolysis of fatty acids in glycerophospholipids (Baburina and Jackowski, 1999). *MPV17* is a mitochondrial inner membrane protein that forms oligomers in lipid bilayers (Sperl and Hagn, 2021), and it has also been shown that low levels of *MPV17* expression are associated with quiescence in energy metabolism. The results indicate that *MPV17* influences the resting energy metabolism by exerting an impact on the mitochondrial respiratory chain and oxidative phosphorylation (OXPHOS) (Jacinto et al., 2021). Diadenosine polyphosphates (e.g., *Ap4A*) are physiologically released compounds, and the roles of their receptors as well as their function as second messengers influencing insulin release have been demonstrated. It has been shown that glucose levels in the blood increase and plasma insulin decreases after *Ap4A* administration in rats (Verspohl et al., 2003a; Verspohl et al., 2003b), and *NUDT2* is thought to be a major factor in maintaining low intracellular *Ap4A* levels (Mclennan et al., 1995; Abdelghany et al., 2001; Carmi-Levy et al., 2008). *ND4L* is involved in the composition of the electron transport chain during oxidative phosphorylation, and dysfunction of this gene leads to metabolic disorders (Dashti et al., 2021), and it is considered to be a major predisposing factor for the development of metabolic syndrome (Perks et al., 2017). In addition, functional annotation of these genes after the KEGG pathway revealed that these genes are enriched in pathways related to lipid deposition such as α-linolenic acid metabolism, linoleic acid metabolism, ether lipid metabolism, and glycerophospholipid metabolism. Based on these results, it was concluded that these four genes play important roles in fat deposition in pigs, and these genes and pathways are not commonly found in traditional analysis methods but are some potential candidates that may affect fat deposition in pigs. This indicates that through machine learning methods were able to find some important information that could not be found by traditional differential analysis methods. This study further confirms the significance of integrating transcriptomic data from different sources (Liu et al., 2022) and shows that machine learning models can provide further technical support for traditional differential analysis methods (Veiner et al., 2022).

There is no single machine learning method that can be applied to all types of samples and different algorithms should be chosen based on the sample characteristics of different studies (Mirza et al., 2019). In this study, after evaluating the performance of both classifiers, it was found that the SVM-RFE model is more accurate than the RF model. Support vector machine algorithm, as a supervised cluster analysis algorithm, has achieved good results in the classification of high-dimensional small sample data with good generalization ability (Cherkassky, 1997), which has been favored by many researchers and is widely used in various fields of research (Zheng Y. et al., 2020; Lin et al., 2021; Shang et al., 2021; Song et al., 2021). The random forest belongs to an integrated algorithm, which itself has better accuracy than most individual algorithms and performs well in many cases (Lam et al., 2021), so it is also widely used in various fields of research (He et al., 2019; Toth et al., 2019; Bi et al., 2020). The choice of the classifier depends on the amount of data and the complexity of the problem, but there are many cases where support vector machines outperform random forests in terms of predictive effectiveness (Caruana and Niculescu-Mizil, 2006). For this study, the number of samples is relatively small and the complexity of the sample information is high, and the SVM-RFE model shows better performance compared to the RF model. This further indicates that different algorithms for different sample characteristics should be chosen, which is the only way to ensure the accuracy of the classification and the reliability of the results.

## Conclusion

This study integrated transcriptomic datasets from different studies to identify important genes by combining traditional gene expression analysis and machine learning methods and finally screened a total of four important genes, *PLA2G6, MPV17, NUDT2*, and *ND4L*. At the same time, some important pathways were identified. This study screened consistent key genes affecting intramuscular fat deposition from different breeds of pigs, providing new reference information for the study of molecular regulatory mechanisms of porcine fat deposition.

## Data Availability

Both original dataset and publicly available datasets were analyzed in this study. This data can be found here: https://www.ncbi.nlm.nih.gov/sra/, accession numbers PRJNA1043865, PRJNA776032, PRJNA302287, PRJNA359473, PRJNA480676, PRJNA695218, PRJNA387276, PRJNA743884 and PRJNA604841.
