# Supplementary material for: Identification of key genes affecting intramuscular fat deposition in pigs using machine learning models
Source: Front Genet. 2025 Jan 6;15:1503148. doi: 10.3389/fgene.2024.1503148 (PMC11743517; doi:10.3389/fgene.2024.1503148)
Supplement: Supplementary file 2 [file Table1.DOCX]

**Supplementary Tables**

Table 1 Sequencing Library Quality Statistics

| Accession number | Clean reads | Clean base | Read length (bp) | GC(%) | Q20(%) |
| --- | --- | --- | --- | --- | --- |
| Ours | 233.52M | 34.80G | 148;148 | 56.94% | 94.38% |
| PRJNA776032 | 1836.60M | 265.30G | 144;144 | 47.25% | 98.39% |
| PRJNA302287 | 417.54M | 41.70G | 99;99 | 51.00% | 98.22% |
| PRJNA359473 | 252.36M | 24.48G | 96;97 | 51.72% | 97.78% |
| PRJNA480676 | 1838.76M | 139.56G | 75;75 | 50.16% | 97.73% |
| PRJNA695218 | 333.90M | 50.04G | 149;149 | 50.58% | 98.08% |
| PRJNA387276 | 555.84M | 78.90G | 141;141 | 51.19% | 97.85% |
| PRJNA743884 | 432.48M | 64.80G | 149;149 | 52.22% | 98.11% |
| PRJNA604841 | 2312.04M | 231.60G | 100;100 | 47.02% | 97.95% |
