# Supplementary figures and images for: Identification of key genes affecting intramuscular fat deposition in pigs using machine learning models
Source: Front Genet. 2025 Jan 6;15:1503148. doi: 10.3389/fgene.2024.1503148 (PMC11743517; doi:10.3389/fgene.2024.1503148)

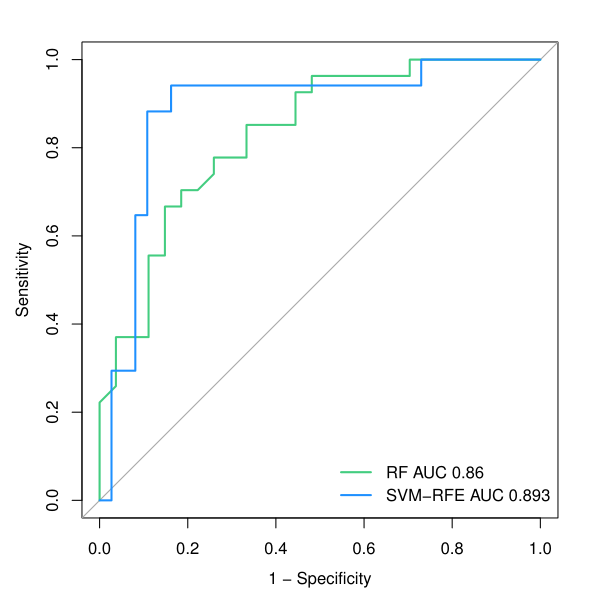

Supplement: Supplementary file 3 [file Image2.TIF]

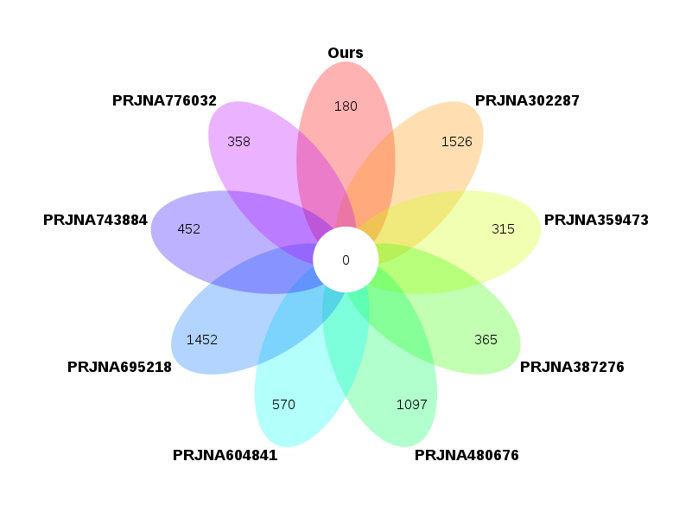

Supplement: Supplementary file 4 [file Image1.TIF]
