# Supplementary material for: Identification of key genes affecting intramuscular fat deposition in pigs using machine learning models
Source: Front Genet. 2025 Jan 6;15:1503148. doi: 10.3389/fgene.2024.1503148 (PMC11743517; doi:10.3389/fgene.2024.1503148)
Supplement: Supplementary file 5 [file Table2.DOCX]

**Supplementary Tables**

Table 2 Mapping rate for each sample

| HIMF | Rates(%) | LIMF | Rates(%) |
| --- | --- | --- | --- |
| SL1 | 94.67% | LL1 | 95.40% |
| SL2 | 94.52% | LL2 | 94.70% |
| SL3 | 94.42% | LL3 | 94.91% |
| SH1 | 94.05% | LH1 | 94.02% |
| SH2 | 94.40% | LH2 | 94.47% |
| SH3 | 94.80% | LH3 | 94.20% |
| SRR16767126 | 97.97% | SRR16767131 | 97.61% |
| SRR16767127 | 97.53% | SRR16767132 | 98.75% |
| SRR16767128 | 97.96% | SRR16767133 | 97.37% |
| SRR16767129 | 97.31% | SRR16767134 | 97.55% |
| SRR16767130 | 97.75% | SRR16767135 | 97.97% |
| SRR2919657 | 96.40% | SRR2919660 | 97.07% |
| SRR2919658 | 96.28% | SRR2919661 | 97.50% |
| SRR2919659 | 97.03% | SRR2919662 | 97.36% |
| SRR5136325 | 95.91% | SRR5136327 | 95.41% |
| SRR5136326 | 95.49% | SRR5136328 | 96.32% |
| SRR7511281 | 98.46% | SRR7511287 | 98.46% |
| SRR7511282 | 97.02% | SRR7511288 | 98.46% |
| SRR7511283 | 97.58% | SRR7511289 | 98.25% |
| SRR7511284 | 97.58% | SRR7511290 | 98.25% |
| SRR7511285 | 97.92% | SRR7511291 | 98.16% |
| SRR7511286 | 97.92% | SRR7511292 | 98.16% |
| SRR13559788 | 96.24% | SRR13559791 | 96.05% |
| SRR13559789 | 96.11% | SRR13559792 | 95.98% |
| SRR13559790 | 96.03% | SRR13559793 | 96.37% |
| SRR5577192 | 97.58% | SRR5577189 | 97.51% |
| SRR5577193 | 96.48% | SRR5577190 | 97.56% |
| SRR5577194 | 97.14% | SRR5577191 | 97.51% |
| SRR15043470 | 97.43% | SRR15043467 | 97.32% |
| SRR15043471 | 97.21% | SRR15043468 | 97.53% |
| SRR15043472 | 97.32% | SRR15043469 | 96.85% |
| SRR11020928 | 98.92% | SRR11020934 | 98.93% |
| SRR11020929 | 98.80% | SRR11020936 | 98.92% |
| SRR11020930 | 98.67% | SRR11020937 | 98.99% |
| SRR11020931 | 98.92% | SRR11020938 | 99.09% |
| SRR11020932 | 98.99% | SRR11020939 | 98.93% |
| SRR11020933 | 99.09% | SRR11020940 | 98.92% |
